# Telehealth for opioid use disorder treatment in low-barrier clinic settings: an exploration of clinician and staff perspectives

**DOI:** 10.1186/s12954-021-00572-7

**Published:** 2021-11-25

**Authors:** Shoshana V. Aronowitz, Eden Engel-Rebitzer, Abby Dolan, Kehinde Oyekanmi, David Mandell, Zachary Meisel, Eugenia South, Margaret Lowenstein

**Affiliations:** 1 University of Pennsylvania School of Nursing, 418 Curie Blvd, Room 419, Philadelphia, PA 19104 USA; 2 University of Pennsylvania Perelman School of Medicine, Philadelphia, PA USA; 3 University of Pennsylvania Center for Emergency Care Policy and Research, Philadelphia, PA USA; 4 Penn Center for Mental Health, University of Pennsylvania, Philadelphia, PA USA

## Abstract

**Background:**

The majority of individuals with opioid use disorder (OUD) face access barriers to evidence-based treatment, and the COVID-19 pandemic has exacerbated the United States (US) opioid overdose crisis. However, the pandemic has also ushered in rapid transitions to telehealth in the USA, including for substance use disorder treatment with buprenorphine. These changes have the potential to mitigate barriers to care or to exacerbate pre-existing treatment inequities. The objective of this study was to qualitatively explore Philadelphia-based low-barrier, harm-reduction oriented, opioid use disorder (OUD) treatment provider perspectives about and experiences with telehealth during the COVID-19 pandemic, and to assess their desire to offer telehealth to patients at their programs in the future.

**Methods:**

We interviewed 22 OUD treatment prescribers and staff working outpatient programs offering OUD treatment with buprenorphine in Philadelphia during July and August 2020. All participants worked at low-barrier treatment programs that provide buprenorphine using a harm reduction-oriented approach and without mandating counseling or other requirements as a condition of treatment. We analyzed the data using thematic content analysis.

**Results:**

Our analysis yielded three themes: 1/ *Easier access for some*: telehealth facilitates care for many patients who have difficulty attending in-person appointments due to logistical and psychological barriers; 2/ *A layered digital divide*: engagement with telehealth can be seriously limited by patients’ access to and comfort with technology; and 3/ *Clinician control:* despite some clinic staff beliefs that patients should have the freedom to choose their treatment modality, patients’ access to treatment via telehealth may hinge on clinician perceptions of patient “stability” rather than patient preferences.

**Conclusions:**

Telehealth may address many access issues, however, barriers to implementation remain, including patient ability and desire to attend healthcare appointments virtually. In addition, the potential for telehealth models to extend OUD care to patients currently underserved by in-person models may partially depend on clinician comfort treating patients deemed “unstable” via this modality. The ability of telehealth to expand access to OUD care for individuals who have previously struggled to engage with in-person care will likely be limited if these patients are not given the opportunity to receive treatment via telehealth.

## Introduction

Prior to the COVID-19 pandemic, the United States was facing a public health crisis of substance use disorders (SUD) and overdose [1, 2]. Despite the existence of effective treatment strategies for opioid use disorder (OUD)—particularly medications for OUD (MOUD) such as buprenorphine and methadone—a minority of impacted individuals receive treatment and there are substantial racial, ethnic, socioeconomic, and geographic disparities in receipt of care [3–5]. The COVID-19 pandemic has only served to exacerbate many of the barriers to accessing care, including social isolation, financial stress, and closures of medical, harm reduction and social services [6–8]. Overdoses have spiked across the country during the pandemic, especially in communities of color [8, 9].

The COVID-19 pandemic has also ushered in rapid transitions to telehealth modalities, including SUD treatment, potentially mitigating some barriers to care. Historically, federal law required an in-person visit for initiation of controlled substances, including MOUDs [10]. In March 2020, the Drug Enforcement Agency (DEA) temporarily lifted requirements for in-person assessment for initial prescriptions of controlled substances, allowing for both buprenorphine induction and maintenance to occur entirely remotely [11]. Telehealth may facilitate equitable access to OUD treatment by addressing geographic and transportation barriers and allowing patients with competing responsibilities like work and childcare to engage in treatment [10, 12]. However, telehealth for OUD care delivery could also worsen pre-existing inequities among people who use drugs (PWUD) who have limited connection to health services, lack of access to technology, and other social challenges that impact their ability to obtain care virtually. As highlighted by the Netherland and Hansen, access to SUD treatment is often multi-tiered. Despite the potential for newer treatments and delivery modalities to engage individuals who struggle the most to access care, it is often wealthier patients with ample resources who benefit from new treatment technologies [13, 14].

Little published research has focused on provision of OUD treatment via telehealth for the most marginalized populations with OUD (i.e.,: individuals experiencing poverty, co-occurring mental health issues, criminal justice involvement, unstable housing, and/or previous struggles to succeed in treatment). Existing work is largely limited to descriptions of low-barrier treatment programs during the pandemic designed to maintain continuity of care and serve the complex needs of this patient population [15–17]. Low-barrier treatment refers to care delivery models tailored to the needs of marginalized PWUD often including flexible, rapid treatment access, a harm reduction-oriented approach, and limited requirements in order to access MOUDs [18]. These programs often serve individuals who have been unable to succeed in programs with more rigid requirements due to ongoing substance use, missed appointments, or lack of engagement with counseling. Little is known about the experiences and perspectives of OUD treatment prescribers and staff in low-barrier treatment settings on pandemic-related shifts to telehealth. In order to ensure that telehealth programs expand access to OUD treatment rather than increase disparities in treatment availability, it is vital to explore how low-barrier, harm reduction oriented programs serving marginalized PWUD have used telehealth during the COVID pandemic and plan to do so in the future. Issues of access to technology are important, however, clinician and program comfort with providing non-traditional treatment modalities to patients often regarded as “unstable” (i.e.,: unhoused, unemployed, or living with additional mental health problems) may represent a larger barrier to telehealth treatment access for marginalized PWUD.

Philadelphia, which has the highest overdose death rate of any large U.S. city [19, 20], is home to several low-barrier, harm reduction oriented programs, which typically include MOUD prescribers (physicians, nurse practitioners, physician assistants) as well as other licensed and unlicensed clinicians and staff (RNs, social workers, peer workers) in service provision [21, 22]. We conducted semi-structured interviews with Philadelphia-based OUD treatment prescribers and staff from low-barrier programs to explore their perspectives about and experiences with the use of this new treatment delivery modality during the COVID-19 pandemic, and to assess their desire to offer telehealth to patients at their programs moving forward.

## Methods

### Participants

In this qualitative descriptive study, we interviewed 22 OUD treatment prescribers and staff (nurse practitioners, physician assistants, physicians, nurses, social workers, and peer workers) working at low-barrier, outpatient OUD treatment programs providing buprenorphine in Philadelphia during July and August 2020. Individuals were eligible if they worked in any of these roles at a low-barrier OUD treatment program in Philadelphia. Although low-barrier care can take many forms, for the purposes of this study, low-barrier programs were defined as those taking a “medication first” approach. All programs offered buprenorphine without requirements for abstinence from opioids or other substances or participation in counseling or behavioral treatment, and most were housed in non-traditional treatment settings, like harm reduction organizations or mobile units. This is in contrast to more traditional treatment models that often have more stringent requirements in order to receive MOUDs. As methadone is much more tightly regulated than buprenorphine in the United States and can rarely be provided at non-traditional sites or via “low-barrier” models [23, 24], we did not include providers and staff at methadone programs in this study.

### Data collection

After approval from the University of Pennsylvania IRB, we recruited participants via social media postings and targeted outreach to treatment program staff known to the study team. We also used snowball sampling to recruit additional participants. Interviews were conducted via BlueJeans teleconferencing software or phone based on participant choice, and were semi-structured, one-on-one, and approximately 30–45 min. All interviews were audio-recoded and transcribed. The design of the interview guide was based on the Social-Ecological Model [25] as we aimed to assess the impact of the switch to telehealth at multiple levels, including the individual, program/clinic, and community levels. The interview guide included questions about participants’ experiences using telehealth, how the transition to telehealth impacted them and how they perceive it impacted their patients, concerns about using telehealth to provide OUD care, how telehealth was integrated into the workflow at their clinics, and their perceptions about how telehealth was being used at other treatment programs across the city. All interviews were conducted by a research team member who herself works as an OUD treatment clinician at a low-barrier program in Philadelphia. She engaged in reflexive journaling [26] throughout the study as a way to remain consciously aware of her positionality with regards to study participants, with whom she shared many experiences. Participants received a $20 VISA giftcard as compensation.

### Data analysis

After transcription and cross-checking, two research team members used NVIVO 1.3.1 software to analyze interview transcript and field note data with thematic analysis methodology. They first read through interviews to become familiar with the content. They then created a preliminary codebook by coding the first three interviews and used this codebook to code the remaining interviews. They met weekly throughout this process to discuss the analysis and refine the codebook. With input from other research team members, the coders then created and named themes based on the codebook.

## Findings

### Participant characteristics

Participants included 16 women, five men, and one non-binary individual. Roles included nurse, physician, physician assistant, social worker, and peer recovery specialist. Mean age was 32.1 years, with an average of 6.2 years of experience working in SUD treatment and/or harm reduction service provision. Participants worked at programs that served primarily, although not exclusively, patients experiencing unstable housing, unemployment, current or previous criminal justice involvement, and co-occuring mental health conditions.

## Results

Our analysis yielded three themes: 1/ *Easier access for some*: telehealth facilitates care for many patients who have difficulty attending in-person appointments due to both logistical and psychological barriers; 2/ *A layered digital divide*: engagement with telehealth can be seriously limited by patients’ access to and comfort with technology; and 3/ *Clinician control:* despite some clinic staff beliefs that patients should have the freedom to choose their treatment modality, patients’ access to treatment via telehealth may hinge on clinician perceptions of patient “stability” rather than patient preferences.

### Easier access for some

Participants universally voiced that telehealth was an important means of improving access to medication and other care for some patients with OUD. Regulatory changes coupled with social distancing guidelines increased prescriber and staff comfort with offering telehealth to patients who previously would be required to come for in-person care. This increased flexibility alleviated logistical barriers to visit attendence that some patients expressed had previously, including transportation challenges and competing responsibilities like work and childcare. One social worker stated:

For the patients who’ve been maintained in care, and that’s the majority of them, by and large they’ve really liked [telehealth]. I think it allows them a lot of flexibility. Some of my patients are parents to younger children and so childcare was always an issue…[Patients] would often tell me they would miss an appointment because they couldn’t get off of work. So, being able to kind of see their provider and see their treatment team without it being so disruptive, I think has been overall positive.

This “silver lining to COVID” applied most commonly to patients who were already retained in treatment, often those who were deemed “stable” and engaged in outside work or other responsibilities. In addition, participants reported that patients sometimes had been reluctant to present in-person for treatment because it involved distressing experiences, such as seeing people or places associated with past drug use. One participant paraphrased a patient who said “listen, just even coming down here for my appointment is a trigger.” Another stated that telehealth allows patients to “just call in if they are feeling like they want to stay away from the neighborhood.”

Participants also voiced that telehealth also improved care for patients who had what participants viewed as fluctuating motivation for treatment, allowing for initiation of medication at the moment a patient was ready. One prescriber stated:

I think the biggest area we need flexibility in is the first visit. I think when patients decide that they are interested in starting buprenorphine there’s a very limited window for thousands of reasons on why that window might not present itself later in the week or later in the month…As long as we’re legally permitted to induct patients via telehealth, I think we should take advantage of the patient being ready in the moment.

For this group of patients—patients who participants deemed less “stable” but who expressed a desire for treatment–telehealth allowed for new or re-engagement in treatment at a time when they felt ready.

### A layered digital divide

Despite the potential to improve access through increased flexibility, the switch to telehealth modalities appeared challenging for marginalized patients with unreliable access to the technologies. At the most basic level, patients did not always have stable phones or internet, making it challenging to connect with them reliably for phone or audiovisual visits. One peer recovery specialist stated: “In the unsheltered population in Philly people just don’t have reliable cell phones often times or a good enough data plan…to do video calls or things like that.” Another prescriber said:

[We’ve had] a lot of challenges getting in touch with people. They’re not available or they’re not available at that time. People’s numbers are in and out. Some individuals only have phones that have Wi-Fi availability, so if they’re not in a Wi-Fi network clearly we can’t connect with them.

Participants also believed that there was substantial variability in patients’ comfort and ability to use different applications or platforms for telehealth visits. One social worker stated:

There are some patients who we just connect via telephone, either because they don’t have the ability to download the apps on their phones because they don’t have smartphones and don’t have computers or because they don’t have the computer literacy to do so.

Some participants reported that their organizations attempted to bridge this digital divide by having prescribers work remotely but having patients and case managers remain in-person. One case manager described:

We obviously have a lot of [patients] that don’t have phones or let alone computers, so I would say like there’s still a good amount of patients who are coming to the building and not participating in telehealth. And when they – I should clarify. So they’re not participating in telehealth on their end, but if they come into the building and do want to speak with a provider then they can engage with it [by using a case manager’s device].

Organizations adapted to support patients’ needs through hybrid approaches, but this still required substantial presence of in-person staff, often the non-prescribing team members who were lower paid or more junior in the organization.

Beyond these clear technology barriers, participants also described a less tangible digital divide, in which patients were physically able to connect with providers for telehealth visits but struggled to adapt to this modality. In some cases, participants described what they believed was a preference for in-person care over virtual visits. One case manager shared a story of a patient who seemed to decline a clinic visit because the prescriber was not physically on-site:

I had somebody come in yesterday and ask to meet with a medical provider. And I said, sure, can you give me one minute…And the person was like, will it be how it was last time, do I have to meet with the person through the screen? And I said, yeah, we have to–that’s the way that the appointment will go. And the person said, no, I’ll just come back later.

Others described patients seemingly having difficulty understanding the nature of care delivered by telephone or audiovisual modalities. In describing the shift to telehealth, another case manager reported:

For me, it’s been a relatively seamless experience. But for the [patients], it has not been. What I’m finding is people are having trouble connecting that they actually still have a provider. Forsome reason, there’s some disconnect with them seeing the provider over the phone. I’ve had multiple people come up to me or express to other staff that they haven’t seen a doctor, when I know that they’ve seen a doctor [via telehealth].

### Clinician control

The last theme was a sense of uncertainty about the future and to what degree practices would shift back toward traditional, in-person approaches. Participants questioned how decisions about telehealth would be made once in-person services resumed at full capacity. Much of the discussion centered around control: whether telehealth care would be offered based on patient or clinician preference. Some participants remained open to offering a hybrid of telehealth and in-person visits based on patients needs or preferences, whether it was to accommodate logistical challenges or simply because it was the patients’ preferred modality. A social worker stated:

No one’s forced to come in if they don’t want to come in, and no one’s forced to have a telephone visit if they really would prefer to come in. We’re not telling people, listen, your visit’s either in person or not at all.

Within this group of participants, there was a sense that telehealth should become part of their service repertoire in a flexible way so that patients could choose their preferred option.

Other participants described approaches that were prescriber or program-driven. A few prescribers preferred to continue telehealth care for their own benefit and because of the flexibility it provided them personally. One said:

I mean it’s definitely more comfortable, just from a personal standpoint, not having that commute and working from home, I kind of enjoy it.

Others expressed preferences for in-person care because they felt they were able to better engage with patients. One prescriber described “hating” telehealth because of the lack of face-to-face connection meant that the quality of depth of care suffered:

I like to connect with my patients in the room, the body language. It’s hard, right? We like can’t give hugs. That’s what we do. We connect with the patients. [Telehealth] was better than nothing, so I’m really grateful that we didn’t have to be completely disconnected to our patients, but it’s not the same.

Ultimately, many participants said they were likely to continue offering telehealth to a subset of patients based on provider perceptions of patient stability and engagement. One said:

Joining a call is a lot easier then showing up in person. And I think that it’s important that patients have a complete investment in their treatment and having a space that is just for them. And I wouldn’t want telehealth to ever be used as an avoidance strategy… I think that that would have to be up to the clinician and the specific patient to target why are you doing telehealth instead of in person…is that actually supportive of your therapy goals or is it not?

Another prescriber stated:

It’s relates to where the person is in terms of their use and recovery. I think my guess and my experience is that for people who are very stable [the switch to telehealth] hasn’t really made much of a ruffle. I think that we’ve been able to call in scripts, keep them going, and as long as no real other life traumas or something that interrupted their recovery came about, they carried on. For people – say I’m gonna go extremes. For people, say, who are just starting, unsheltered, very unstable, it doesn’t – it seems that that’s been very challenging… it is very challenging with people who are marginalized in any way.

This left clinicians and other staff in a position of deciding who was able to receive different types of services, which some found uncomfortable. One case manager described the situation where prescribers work remotely while social workers and patients meet in person:

It’s emblematic of oftentimes the role of social work healthcare providers in recovery treatment, which is to be the gatekeeper, sort of, to care. And in those situations where the provider asks like ‘how do they look, how are they doing?’…what I would prefer would be for the provider and the participant to just talk and come to that conclusion. But I’m recognizing now just speaking about it that that is just passing the gatekeeper role from me to the provider.

Ultimately, the tension between provider versus patient control was something that many participants were wrestling with. Some were relieved by reducing aspects of surveillance such as frequent urine drug screenings, whereas others felt that without the tangible and intangible information gathered at in-person visits, they were less able to provide quality care:

For patients that you don’t know – so for example there are a lot of individuals because they lost – they became unemployed, lost their health insurance, and now they’re scheduling these new patient visits and they’re all telehealth at our clinic. That’s really hard because you don’t have the baseline of knowing the patient to get a sense of am I getting, am I asking the right questions? Or are we building rapport here? It’s kinda harder to do over the phone.

For many, the future remained uncertain, but there was excitement on the part of some participants about the possibility that the greater flexibility and patient-centeredness brought on by COVID would remain as the pandemic waned. One social worker with lived experience of SUD stated:

That’s my hope. I mean I would love for it to be person-driven, right, like what the person feels is best versus what’s like decided by a provider. And I say that as a non-provider, right, like just as like a social worker, as a person in recovery who’s received services in a million different ways. I would love if it could be as simple as the person says I prefer to do telemedicine visits versus coming in person, that that was what they got.

## Discussion

Telehealth presents an opportunity to expand access to OUD care with buprenorphine for many individuals in the United States by addressing multiple barriers including geography, transportation, and conflicting responsibilities. Participants in our study shared many examples of patients they thought could easily engage in treatment virtually. This benefit has been highlighted in previous studies and commentaries about the use of telehealth for SUD specifically and for other types of care [10, 27, 28]. Talal et al.’s adaptation of the Sociotechnical System model for delivery of patient-centered OUD and hepatitis C care is a useful framework for considering our findings, as many telehealth models and approaches are not tailored to programs serving marginalized populations [29].

Questions remain about how to support marginalized patients in telehealth engagement. Our findings regarding the struggle to ensure that patients have the technology needed to complete telehealth visits echo the concerns of others pushing for expanded access to telehealth for SUD treatment as well as other types of care [30–32]. While phone and internet use is ubiquitous across socioeconomic groups including unstably housed individuals, low-income and unstably housed patients are more likely than wealthier patients to have interruptions in access to digital technologies (due to changing phone numbers or trouble paying bills) [33]. This disparity can effectively shut off access to telehealth for patients who cannot consistently afford the technology. Interventions that provide phones and free access to consistent high-quality data plans may target this barrier.

Our results highlight an additional layer of the “digital divide” that cannot easily be addressed with increased access to devices. We found that some patients may decline to engage with telehealth because they perhaps do not like the modality (“*do I have to meet with the person through the screen?…I’ll come back later”)* or that some patients may engage but not accept that they have completed a visit with a healthcare provider (“*I’ve had multiple people come up to me or express to other staff that they haven’t seen a doctor, when I know that they’ve seen a doctor [*via* telehealth]”).* It is possible that this reaction to telehealth may be related to an individual’s level of engagement with technology more broadly. Although diverse economies and institutions—including drug markets and sex work—have embraced digital technologies in order to persevere in the face of COVID-19 physical distancing requirements, marginalized PWUD engaged in drug purchasing and selling or sex work may not have the option to move their activities online and have continued to conduct their appointments and business in-person [34–36]. Virtual healthcare appointments may be less palatable or acceptable to those whose lives and livelihoods remained otherwise mostly or entirely in-person compared to individuals who became accustomed to a digital world. Talal et al.’s adaptation of the Sociotechnical System model addresses how lack of comfort with and trust of technology might impact marginalized patients’ engagement with telehealth, pointing out that some patients may fear that telehealth encounters are less private than in-person encounters or that their health information may not be protected. To engage new and/or skeptical patients, Talal et al. suggest using testimonials of other patients who have successfully received OUD care via telehealth [29].

Finally, our findings highlight the tension at play between patient versus clinician preferences about care delivery. Studies of methadone clinics have found that while loosened federal restrictions associated with the COVID-19 pandemic resulted in increased take-home doses for many patients, this varied by clinic [37, 38], suggesting that prescribers and program staff likely used discretion in deciding which patients could receive more flexible care. Additionally, patient “stability”—determined partially by the absence of ongoing substance use and other factors like “confidence” that a patient would take their medication as prescribed—impacted the likelihood that methadone programs would allow for take-home dosing [39]. While some participants in our study, namely, social workers and case managers, voiced support for models that centered patient choice in care delivery, others shared their own dislike of telehealth and concerns about the quality of care delivered, especially for patients they deemed “unstable.”

Urine drug screening, although viewed by many clinicians as an important tool to measure patient engagement in treatment, is more difficult to administer via telehealth. A urine drug screen that is negative for buprenorphine may spur a conversation in which a clinician learns that a patient consistently loses their medication, a situation that may be in part addressed with shorter prescriptions; if a prescriber suspects that a patient is selling or sharing some of their medication, the patient may be discharged from a program [40]. Without urine drug screenings, many clinicians will likely have concerns about whether patients are taking buprenorphine as prescribed. It is unclear from our findings how clinicians make determinations about which patients or situations warrant urine drug screening, although previous research focused on urine drug testing for opioid analgesic therapy and in obstetrical care suggests that patient race may be one factor informing testing, with Black patients more likely to undergo screening than white patients [41–43]. Other patient factors, including housing, employment, and financial situation may also impact clinicians’ desire to conduct in-person visits and/or urine drug screening. While a hybrid telehealth model involving an in-person case manager and a remote prescriber would address this issue (the case manager can collect urine specimens or other laboratory results, as outlined by Talal et al.), this type of model does not address the many barriers that patients may face to presenting to a clinic in-person, and, in the context of the COVID-19 pandemic, requires that the patient and case manager risk potential exposure to the virus. A system involving mailed urine drug screen cups might work well for some patients, although it would require a mailing address—a barrier for many unstably housed patients. Participants in our study did not voice concern about undetected ongoing substance use without regular urine drug screening, but this is likely because the programs where they worked were explicitly tolerant of ongoing use, unlike many other programs.

A strength of telehealth approaches may be the ability to serve patients who have struggled to engage with in-person care models. However, these same patients may be those that clinicians believe are too “unstable” or poorly engaged to qualify for virtual visits. More research is needed to explore how determinations of stability versus instability are made, clinicians’ comfort and willingness to provide this type of care to patients regarded as “unstable,” and how clinicians make determinations about which patients are elibigle to receive OUD treatment with buprenorphine via telehealth.

This research has several important limitations. These findings may not be generalizable to the experiences and perspectives of low-barrier treatment program staff outside of Philadelphia. Given that many countries take a more progressive approach to substance use and substance use disorder treatment, these results may not be relevant to providers and patients in countries where OUD treatment is less tightly regulated. Since this study was focused on clinicians and staff, our findings may not accurately reflect the perspectives of program patients, and future studies should involve interviews with PWUD about their experiences with OUD treatment via telehealth.

## Conclusion

Opioid overdose death rates continue to rise across the USA, and it is clear that previous efforts have not been sufficient to stem the crisis. The COVID-19 pandemic offered an opportunity to address OUD treatment access barriers by allowing for expanded use of telehealth. Our qualitative study of staff working with marginalized patients at low-barrier treatment programs in Philadelphia highlights numerous access issues potentially addressed by telehealth approaches as well as barriers to implementation, including patient ability and desire to attend healthcare appointments virtually. Assuming that regulatory changes allowing for telehealth OUD treatment persist, policy should support interventions that increase access to the technologies needed to participate in telehealth among marginalized patients, although many patients may continue to prefer in-person visits. Perhaps most importantly, the potential for telehealth models to extend OUD care to patients currently underserved by in-person models may depend on clinician comfort treating patients deemed “unstable.” Support for harm reduction oriented telehealth provision, including clinician training and policy explicitly endorsing patient choice about care modality, may help clinicians feel more comfortable offering services guided by patient preference.

## Data Availability

In order to protect the confidentiality of participants, interview transcripts are not pubically available.
